# METTL3-mediated mRNA *N*^6^-methyladenosine is required for oocyte and follicle development in mice

**DOI:** 10.1038/s41419-021-04272-9

**Published:** 2021-10-23

**Authors:** Haiyuan Mu, Ting Zhang, Ying Yang, Danru Zhang, Jie Gao, Junhong Li, Liang Yue, Dengfeng Gao, Bingbo Shi, Yue Han, Liang Zhong, Xinze Chen, Zhen-Bo Wang, Zhen Lin, Ming-Han Tong, Qing-Yuan Sun, Yun-Gui Yang, Jianyong Han

**Affiliations:** 1 State Key Laboratory for Agrobiotechnology, College of Biological Sciences, China Agricultural University, Beijing, 100193 China; 2 Key Laboratory of Genomic and Precision Medicine, Collaborative Innovation Center of Genetics and Development, College of Future Technology, Beijing Institute of Genomics, Chinese Academy of Sciences, Beijing, 100101 China; 3 China National Center for Bioinformation, Beijing, 100101 China; 4 University of Chinese Academy of Sciences, Beijing, 100049 China; 5 Institute of Stem Cell and Regeneration, Chinese Academy of Sciences, Beijing, 100101 China; 6 Hebei Provincial Key Laboratory of Basic Medicine for Diabetes, The Shijiazhuang Second Hospital, Shijiazhuang, Hebei, 050051 China; 7 State Key Laboratory of Molecular Biology, Shanghai Key Laboratory of Molecular Andrology, CAS Center for Excellence in Molecular Cell Science, Shanghai Institute of Biochemistry and Cell Biology, Chinese Academy of Sciences, University of Chinese Academy of Sciences, Shanghai, 200031 China; 8 State Key Laboratory of Stem Cell and Reproductive Biology, Institute of Zoology, Chinese Academy of Sciences, Beijing, China; 9 Fertility Preservation Lab, Reproductive Medicine Center, Guangdong Second Provincial General Hospital, Guangzhou, 510317 China

**Keywords:** Oogenesis, Infertility

## Abstract

Proper follicle development is very important for the production of mature oocytes, which is essential for the maintenance of female fertility. This complex biological process requires precise gene regulation. The most abundant modification of mRNA, *N*^6^-methyladenosine (m^6^A), is involved in many RNA metabolism processes, including RNA splicing, translation, stability, and degradation. Here, we report that m^6^A plays essential roles during oocyte and follicle development. Oocyte-specific inactivation of the key m^6^A methyltransferase *Mettl3* with *Gdf9*-Cre caused DNA damage accumulation in oocytes, defective follicle development, and abnormal ovulation. Mechanistically, combined RNA-seq and m^6^A methylated RNA immunoprecipitation sequencing (MeRIP-seq) data from oocytes revealed, that we found METTL3 targets *Itsn2* for m^6^A modification and then enhances its stability to influence the oocytes meiosis. Taken together, our findings highlight the crucial roles of mRNA m^6^A modification in follicle development and coordination of RNA stabilization during oocyte growth.

## Introduction

In mice, a few days after birth, a restricted number of oocytes within primordial follicles serve as the source of mature eggs for reproduction [1]. Through follicle recruitment or activation, resting primordial follicles are recruited into the growing follicle stage featuring oocyte growth and proliferation of somatic granulosa cells [2]. At this stage, oocytes grow rapidly with transcription of a large number of mRNAs that are crucial for subsequent oocyte meiotic maturation and early embryo development. When the oocytes reach the fully-grown germinal vesicle (GV) stage, the maternal transcriptome is completed along with the shutdown of transcription [3]. Throughout the follicle developmental process, oocytes are arrested in the prophase of meiosis I. At puberty, the oocytes resume and complete meiosis I under the stimulation of gonadotropin hormones and are then arrested in metaphase II (MII) until fertilization occurs [4].

Due to the transcriptional silencing of the maternal genome in GV oocytes, normal oocyte meiotic maturation is supported only by early-expressed or stored maternal mRNAs in oocytes. Thus, the maintenance of maternal mRNAs throughout the early developmental stages appears particularly critical. Recent studies have shown that serial epigenetic events participate in process that regulate the composition of oogenesis-associated maternal mRNAs, including DNA methylation [5, 6], histone modifications [7, 8], chromatin remodeling [9, 10], and chromosome stability [11]. Furthermore, the posttranscriptional regulation of poly(A) tail length and RNA modifications has also been validated to play an important role in determining the density of maternal mRNAs [12], suggesting the involvement of a complex regulation mechanism in oocyte development.

In mammals, *N*^*6*^-methyladenosine (m^6^A) is the most abundant internal modification of mRNAs and is involved in many critical aspects of RNA metabolism, including transcription [13], splicing [14, 15], mRNA stability [16, 17], mRNA decay [18, 19], mRNA export [20–22], and translation [23, 24]. The formation of m^6^A is catalyzed by a multicomponent methyltransferase complex including methyltransferase-like 3 (METTL3) [25], methyltransferase-like 14 (METTL14) [26], Wilms tumor 1 associated protein (WTAP) [27], and other proteins. m^6^A is a reversible modification that can be erased by the demethylases fat mass and obesity-associated factor (FTO) [28] and AlkB homolog 5 (ALKBH5) [22] and can be recognized by YTH-domain-containing family proteins [19, 29–31], insulin-like growth factor 2 (IGF2), mRNA-binding proteins [16, 32], and heterogeneous nuclear ribonucleoproteins (HNRNPs) [33] to regulate different developmental processes. Some m^6^A-associated proteins have been reported to participate in oocyte and early embryo development by regulating the turnover of maternal mRNAs, including KIAA1429 and YTHDC1, which regulate maternal mRNA splicing in the GV stage [34, 35], and YTHDF2, which regulates maternal mRNA decay in the MII stage [29]. Recently, IGF2BP2 and IGF2BP3 have also been shown to participate in stabilizing maternal mRNAs for early embryo development in mouse and zebrafish, respectively [36, 37]. However, these studies mainly focused on the functions of non-core m^6^A writer or m^6^A readers in regulating partial maternal mRNAs, the global function of m^6^A in follicle development and oocyte maturation still remains elusive. As the core subunit of the m^6^A methyltransferase complex, METTL3 has usually been used to evaluate the global function of m^6^A both in vivo and in vitro [38, 39]. Nowadays, METTL3-mediated m^6^A has been shown to modulate spermatogenesis [40–42], postimplantation embryonic development [43], sex determination [14, 15], and human diseases [44]. In addition, by knocking down *Mettl3* in GV oocytes from mice, oocyte maturation and early embryo development displayed defects probably due to disrupting mRNA degradation [45]. However, owing to the embryonic lethality of *Mettl3* knockout mice, the in vivo function of *Mettl3* in follicle development remains unknown.

Here, we investigated how m^6^A modification specifically regulates follicle development and ovulation. Our results demonstrated that oocyte-specific inactivation of *Mettl3* with *Gdf9*-Cre causes defective follicle development and infertility. Mechanistically, we found that METTL3-mediated m^6^A modification regulates the stabilization of *Itsn2* at the GV stage and then influences the resumption of meiosis during oocyte development.

## Results

### METTL3 is expressed during follicle development and is required for female fertility

**Fig. 1:**
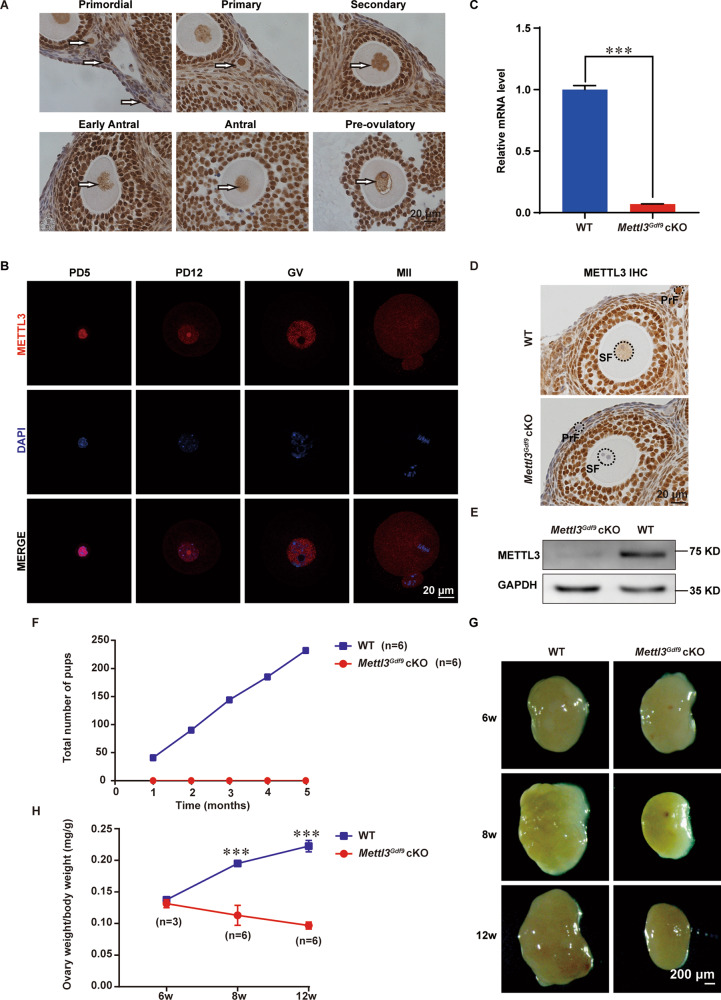
METTL3 is required for female fertility. **A** Immunohistochemistry of METTL3 in 3-week-old mouse ovaries at different follicle stages with PMSG injection. The arrows indicate oocytes at different follicle stages. Scale bar, 20 μm. **B** Confocal immunofluorescence images of oocytes from wild-type mice stained with METTL3 antibody (red) and DAPI (blue), as indicated. PD5 postnatal days 5, PD12 postnatal days 12, GV germinal vesicle, MII metaphase II. Scale bar, 20 μm. **C** qRT-PCR analysis of *Mettl3* mRNA levels in oocytes from 3-week-old WT and *Mettl3*^*Gdf9*^ cKO females. The relative mRNA level of *Mettl3* in WT oocytes was set to 1.0. ***p < 0.001 by two-tailed Student’s *t*-test. Data represent the mean ± SEM (*n* = 3). **D** Immunohistochemistry for METTL3 in ovary sections from WT and *Mettl3*^*Gdf9*^ cKO mice. Primordial (PrF) and secondary (SF) follicle stages are indicated. Scale bar, 20 μm. **E** Immunoblotting analysis of METTL3 protein level in oocytes of WT and Mettl3^*Gdf9*^ cKO mice. GAPDH was used as an internal control. One hundred germinal vesicle oocytes were used for each lane of the blots. **F** Cumulative numbers of pups born from six pairs of WT and *Mettl3*^*Gdf9*^ cKO female mice for 5 months. **G** Representative images of ovaries from 6-week-old, 8-week-old, and 12-week-old female mice. Scale bar, 200 μm. **H** Ratio of ovary weight to body weight of 6-week-old, 8-week-old, and 12-week-old female mice. 6-week-old, *n* = 3; 8-week-old, *n* = 6; 12-week-old, *n* = 6. n.s., *p* > 0.05; ***p < 0.001 by two-tailed Student’s *t*-test. Data represent the mean ± SEM.

To determine the roles of m^6^A in follicle development, we first examined whether METTL3 is expressed in mouse ovaries. The immunohistochemistry results showed that METTL3 was expressed at all stages of folliculogenesis and was mainly located in the oocyte nuclei and granulosa cells (Fig. 1A). Further detection at the single-cell level using immunofluorescence showed that the METTL3 protein was indeed located in the oocyte nucleus at postnatal day (PD) 5, PD12, and the GV stage; however, due to meiotic nuclear division, the METTL3 signal was uniformly dispersed in the oocytes at the MII stage (Fig. 1B). The high abundance and relatively dynamic distribution of METTL3 during the whole process of oocyte development suggest that METTL3 might play a role in the regulation of oocyte competence and maturation. To understand the in vivo functions of *Mettl3* in female reproduction, we generated *Mettl3*^*flox/flox*^;*Gdf9*-Cre (referred to as *Mettl3*^*Gdf9*^ cKO) mice by using *Gdf9*-Cre, which mediates Cre recombinase expression in mouse oocytes at the primordial stage, to knock out *Mettl3* specifically in oocytes [46]. qRT-PCR confirmed that oocytes from *Mettl3*^*Gdf9*^ cKO mice had a negligible expression of *Mettl3* mRNA as compared to *Mettl3*^*flox/flox*^ (referred to as WT) mice (Fig. 1C). Additionally, the immunohistochemistry and immunoblotting results showed that METTL3 protein was nearly undetectable in the oocytes of *Mettl3*^*Gdf9*^ cKO ovaries (Fig. 1D, E), suggesting the successful oocyte-specific knockout of *Mettl3*.

Next, to detect the effect of *Mettl3* knockout on female fertility, we randomly selected six pairs of 6-week-old WT and *Mettl3*^*Gdf9*^ cKO female mice, which were bred to WT male mice for 5 months. Six WT females produced a total of 232 pups, whereas the six *Mettl3*^*Gdf9*^ cKO females produced no offspring (Fig. 1F). Next, we analyzed ovaries obtained at 6 weeks, 8 weeks, and 12 weeks. The sizes of the ovaries and the ovary-to-body weight ratios of *Mettl3*^*Gdf9*^ cKO mice were not significantly different from those of WT mice at 6 weeks, but they were significantly smaller than those of WT mice at 8 weeks. The difference in the ovary-to-body weight ratio between *Mettl3*^*Gdf9*^ cKO and WT mice at 12 weeks was more significant than that at 8 weeks; moreover, the ovary surface was smooth at 12 weeks, with almost no obvious follicle structure (Fig. 1G, H). Together, these results demonstrate that *Mettl3* in oocytes is essential for female fertility.

### *Mettl3*^*Gdf9*^ cKO mice display follicle development defects

**Fig. 2:**
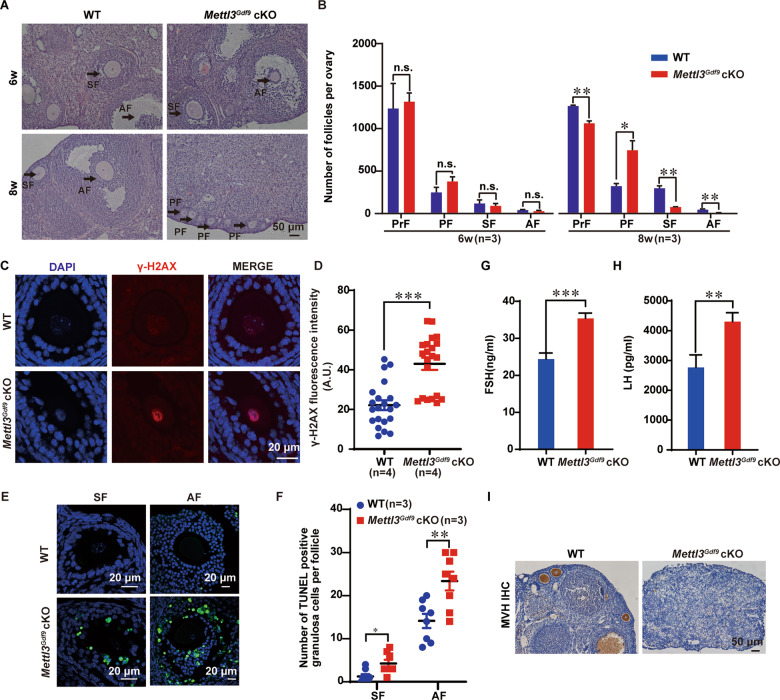
*Mettl3*^*Gdf9*^ cKO mice display follicle developmental defects and POF. **A** Representative ovarian histology of 6-week-old, 8-week-old WT, and *Mettl3*^*Gdf9*^ cKO mice. The primary, secondary, and antral (indicated with PF, SF, and AF, respectively) follicle stages are indicated by the black arrow. Scale bar, 50 μm. **B** Quantification of the numbers of different types of follicles in ovaries from 6-week-old, 8-week-old WT, and *Mettl3*^*Gdf9*^ cKO mice. Primordial, primary, secondary, and antral follicles (indicated with PrF, PF, SF, and AF, respectively) were counted. n.s., *p* > 0.05; *p < 0.05; **p < 0.01 by two-tailed Student’s *t*-test. Data represent the mean ± SEM (*n* = 3). **C** Confocal immunofluorescence staining with γH2AX antibody (red) and DAPI (blue) in ovary sections from 6-week-old WT and *Mettl3*^*Gdf9*^ cKO mouse ovaries. Scale bars, 20 μm. **D** Graph showing the quantification of γ-H2AX staining. ***p < 0.001 by two-tailed Student’s *t*-test. Data represent the mean ± SEM (*n* = 4). **E** Detection of apoptosis in granulosa cells by TUNEL kit performed in paraffin sections at different stages of follicle development of 6-week-old WT and *Mettl3*^*Gdf9*^ cKO mouse ovaries. Secondary and antral follicles (indicated with SF and AF, respectively) were counted. Scale bars, 20 μm. **F** Graph showing the quantification of the number of TUNEL positive granulosa cells at different follicle stages. *p < 0.05; **p < 0.01 by two-tailed Student’s *t*-test. Data represent the mean ± SEM (*n* = 3). **G**, **H** FSH and LH levels of WT and *Mettl3*^*Gdf9*^ cKO mice. Serum samples were collected from female mice of both genotypes (12 to 20 weeks old) and were killed for measurement of FSH and LH levels. FSH: WT, *n* = 5; *Mettl3*^*Gdf9*^ cKO, *n* = 9. LH: WT, *n* = 8; *Mettl3*^*Gdf9*^ cKO, *n* = 9. **p < 0.01; ***p < 0.001 by two-tailed Student’s *t*-test. Data represent the mean ± SEM. **I** Immunohistochemistry with MVH antibody in ovary sections from 24-week-old WT and *Mettl3*^*Gdf9*^ cKO mice. Scale bar, 50 μm.

To investigate the phenotype after *Mettl3* knockout during follicle development, we performed hematoxylin-eosin (HE) staining on ovaries from 6-week-old, 8-week-old females and counted the number of follicles at each stage. In 6-week-old females, there was no significant difference in the number of follicles in each stage. However, in 8-week-old females, there were more primary follicles and fewer primordial, secondary, and antral follicles in the *Mettl3*^*Gdf9*^ cKO ovaries than in the WT ovaries (Fig. 2A, B), demonstrating that METTL3 was not necessary for the transition of primordial follicles to the activated growing follicle stage, but mainly functions in the process of growing follicle development.

The defective follicle development in *Mettl3*^*Gdf9*^ cKO mice suggested that *Mettl3*-deficient oocytes might undergo apoptosis. As DNA damage is a major inducer of apoptosis, we assessed potential DNA damage in oocytes through immunofluorescence assays for phosphorylated histone H2AX (γ-H2AX), a widely used marker gene for DNA double-strand breaks (DSBs) [47]. The results showed that more DSBs were produced in the secondary follicles of the *Mettl3*^*Gdf9*^ cKO mice than in those of the WT mice (Fig. 2C, D). Moreover, we also performed terminal deoxynucleotidyl transferase dUTP nick end labeling (TUNEL) staining to validate this finding. Indeed, *Mettl3*^*Gdf9*^ cKO mice showed a significantly higher apoptosis signal in secondary and antral follicle than WT mice (Fig. 2E, F).

The levels of follicle-stimulating hormone (FSH) and luteinizing hormone (LH) are two important parameters for the assessment of premature ovarian failure (POF) [48]. An enzyme-linked immunosorbent assay (ELISA) showed that the levels of FSH (Fig. 2G) and LH (Fig. 2H) were higher in the serum of 12- to 20-week-old *Mettl3*^*Gdf9*^ cKO mice than in that of WT mice. Moreover, immunohistochemistry for MVH (an oocyte marker) in 24-week-old ovaries indicated that oocytes were absent in *Mettl3*^*Gdf9*^ cKO ovaries, which lead to POF (Fig. 2I). Thus, METTL3 is indispensable for follicle development because it preserves oocyte survival.

### *Mettl3*^*Gdf9*^ cKO oocytes fail to resume meiosis

**Fig. 3:**
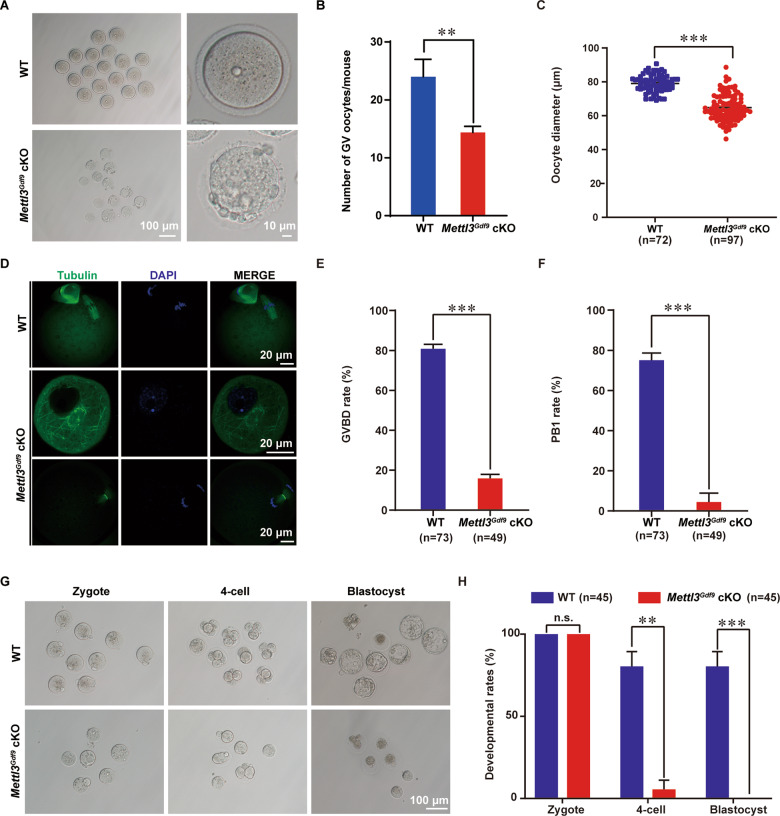
METTL3 is required for oocyte meiotic maturation and early zygotic development. **A** Representative images showing GV stage oocyte from 6-week-old WT and *Mettl3*^*Gdf9*^ cKO females. Left column: scale bar, 100 μm; right column: scale bar, 10 μm. **B**, **C** Mean number and diameter of GV stage oocytes obtained per mouse after priming with PMSG. WT, *n* = 72; *Mettl3*^*Gdf9*^ cKO, *n* = 97. **p < 0.01; ***p < 0.001 by two-tailed Student’s *t*-test. Data represent the mean ± SEM. **D** Confocal immunofluorescence with α-Tubulin antibody (green) and DAPI (blue) for oocytes from 6-week-old WT and *Mettl3*^*Gdf9*^ cKO mice after PMSG and HCG injection. Scale bars, 20 μm. **E**, **F** GVBD percentages and first polar body (PB1) rates for oocytes from 6-week-old WT and *Mettl3*^*Gdf9*^ cKO mice after PMSG and HCG injection. WT, *n* = 73; *Mettl3*^*Gdf9*^ cKO, *n* = 49. ***p < 0.001 by two-tailed Student’s *t*-test. Data represent the mean ± SEM. **G** Representative images of embryos collected from WT and *Mettl3*^*Gdf9*^ cKO mice at the indicated time points after natural ovulation. Scale bar, 100 μm. **H** Rates of a zygote, four-cell, and blastocyst formation by ovulated WT and *Mettl3*^*Gdf9*^ cKO oocytes after culture in KSOM medium. WT, *n* = 45; *Mettl3*^*Gdf9*^ cKO, *n* = 45. n.s., *p* > 0.05; **p < 0.01; ***p < 0.001 by two-tailed Student’s *t*-test. Data represent the mean ± SEM.

To further explore the potential mechanism leading to follicle development defects after *Mettl3* deletion, we collected GV oocytes from 6-week-old mice after pregnant mare serum gonadotropin (PMSG) treatment. The experimental results showed that the number of GV oocytes in *Mettl3*^*Gdf9*^ cKO mice was significantly reduced, and the oocyte diameter also became obviously smaller compared with WT mice (Fig. 3A–C).

It has been reported that meiotic errors can reduce oocyte developmental competence [49]. We thus investigated the influence of *Mettl3* knockout on meiosis and performed immunofluorescence staining for Tubulin to detect spindle formation. We found that most of the oocytes from *Mettl3*^*Gdf9*^ cKO could not undergo germinal vesicle breakdown (GVBD); with only a small number of oocytes reached the end of meiosis I, and almost no first polar body exclusion occurred, suggesting that no mature MII oocytes were produced in *Mettl3*^*Gdf9*^ cKO mice (Fig. 3D–F). These results indicated that the resumption of meiosis in *Mettl3*^*Gdf9*^ cKO oocytes was defective, which influenced the maturation of the oocytes. In addition, the results of subsequent assays examining the developmental competence of ovulated oocytes showed that there was no significant difference in ovulation number between 6-week-old *Mettl3*^*Gdf9*^ cKO and WT mice. However, most of the oocytes from *Mettl3*^*Gdf9*^ cKO mice could not be fertilized to form zygotes or develop beyond the four-cell embryo stage (Fig. 3G, H). But almost no zygotes could be obtained from 8-week-old *Mettl3*^*Gdf9*^ cKO mice compared with WT mice by natural ovulation (Fig. S1A, B). These results suggested that oocyte developmental competence was severely impaired.

### METTL3-mediated m^6^A maintains maternal mRNA stability in oocytes

**Fig. 4:**
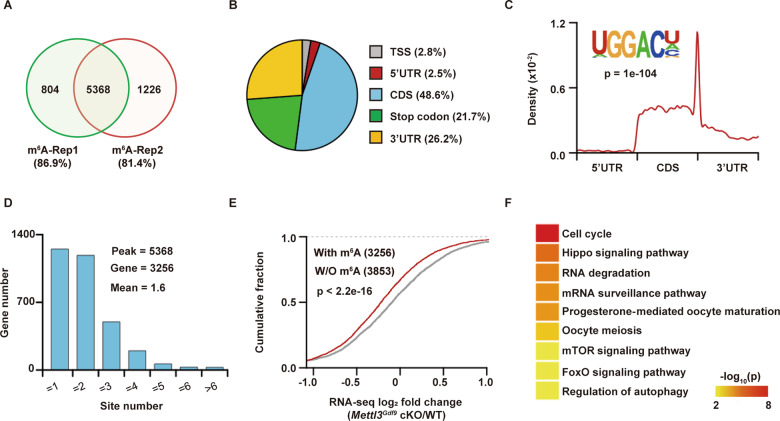
Characteristics of METTL3-mediated m^6^A at the GV stage. **A** Venn diagram showing the overlay of m^6^A peaks in transcripts between two independent biological replicates. **B** Pie plot showing the percentages of m^6^A peaks on distinct mRNA segments: the TSS, 5′ untranslated region (5′UTR), CDS, stop codon, and 3′ untranslated region (3′UTR). **C** The m^6^A motif of GV oocytes enriched with HOMER (inner panel). The distribution pattern displays the position density of m^6^A across the 5′UTR, CDS, and 3′UTR of mRNAs (outer panel). **D** Bar plot showing the numbers of transcripts with different numbers of m^6^A peaks. **E** Cumulative distribution showing the differences (log_2_ (fold change) values) in transcript expression between *Mettl3*^*Gdf9*^ cKO and WT oocytes. The transcripts were classified as m^6^A and non-m^6^A by MeRIP-seq. The *p* value was determined with the two-sided Kolmogorov–Smirnov test. **F** Heatmap showing the significantly enriched KEGG pathways regulated by transcripts with m^6^A modification.

As the core subunit of the m^6^A methyltransferase complex, METTL3 mainly mediates m^6^A formation on mammalian mRNAs. To identify potential maternal mRNA targets regulated by METTL3, we first conducted the m^6^A methylated RNA immunoprecipitation sequencing (MeRIP-seq) assay on GV oocytes. More than 81% (5368) overlapping m^6^A peaks were detected in two independent biological replicates (Fig. 4A and Table S1); these peaks corresponded to 3256 (45.8%) maternal mRNAs, indicating that m^6^A plays a more important role in regulating the density of oocyte maternal mRNAs than previously reported. Consistent with the findings of previous studies, the m^6^A peaks detected in GV oocytes were significantly enriched in the GGACH motif (H = A/C/U) and were abundant in the coding region (CDS), in the 3′ untranslated region (3′UTR), and near the stop codon (Fig. 4B, C). And 90.1% of the methylated mRNAs contained fewer than four peaks, with an average of two peaks per mRNA (Fig. 4D) in this stage.

To further investigate the influence of *Mettl3* deficiency on maternal mRNA abundance, we compared the transcriptomes between WT and *Mettl3*^*Gdf9*^ cKO oocytes at the GV stage based on RNA-seq data. We found that the abundance of maternal mRNAs with m^6^A peaks was significantly decreased than that of maternal mRNAs without m^6^A peaks upon *Mettl3* knockout (Fig. 4E), suggesting that maternal mRNAs with m^6^A modification are preferentially stabilized in the GV stage. In addition, functional enrichment analysis showed that m^6^A-modified maternal mRNAs preferentially participate in the regulation of the cell cycle, oocyte meiosis, and RNA degradation (Fig. 4F).

**Fig. 5:**
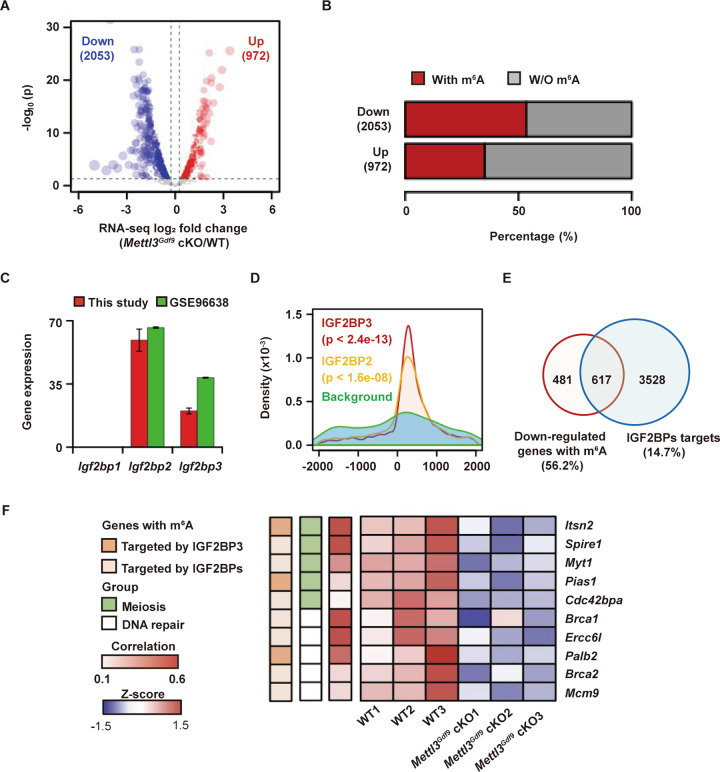
METTL3-mediated m^6^A regulates RNA stability to affect oocyte maturation. **A** Volcano plot showing the expression differences for target transcripts under *Mettl3*^*Gdf9*^ cKO. Transcripts with average RPKM values >3 in WT, |log_2_ fold change| values > log_2_ (1.2), and *p* values <0.05 as determined by DESeq2 were regarded as significantly dysregulated transcripts. The numbers of significantly downregulated (blue) and upregulated (red) transcripts are shown. The vertical dashed lines indicate the cutoff of |log_2_ fold change| = |log_2_ (1.2)|, and the horizontal dashed lines indicate the cutoff of *p* = 0.05. **B** Bar plot showing the ratios of transcripts with m^6^A modification among downregulated transcripts and upregulated transcripts under *Mettl3* knockout. **C** Bar plot showing the expression of *Igf2bp1, Igf2bp2*, and *Igf2bp3* in GV oocytes, as determined by RNA-seq in this study and in the GSE96638 dataset. The data were shown as the mean ± SEM of two independent experiments for this study and 26 independent experiments for the GSE96638 dataset. **D** Density plot showing the distance between IGF2BP2/3 peaks identified by eCLIP (GSE78509) in hESCs and m^6^A peaks identified by MeRIP-seq in this study. The background was obtained by randomly shuffling IGF2BP peaks using BEDTools’ shuffleBed tool. The *p* value was determined by the two-sided Kolmogorov–Smirnov test. **E** Venn diagram showing the overlay between downregulated expressed transcripts with m^6^A peaks and IGF2BP2/3 target transcripts. **F** Heatmap displaying the transcript abundance in WT and *Mettl3*^*Gdf9*^ cKO oocytes. Transcripts with m^6^A modifications targeted by IGF2BPs are labeled brown, transcripts involved in meiosis or DNA repair are labeled in green or white, and the expression correlation between transcripts and *Mettl3* is shown in red.

Consistent with the MeRIP-seq data, the RNA-seq data revealed that 2053 maternal transcripts were significantly downregulated upon *Mettl3* knockout, approximately two-fold more than those that were upregulated (Fig. 5A and Table S2). Functional enrichment analysis showed that oocyte-specific knockout of *Mettl3* globally reduced the abundance of cell cycle-, oocyte maturation-, and meiosis-related maternal mRNAs (Fig. S2A and Table S3). In addition, we identified 1098 (53.5%) downregulated maternal mRNAs with m^6^A modification, significantly more than the 342 (35.2%) upregulated mRNAs (p < 2.2e-16, Chi-squared test, Fig. 5B). The downregulated maternal mRNAs with m^6^A modification were also significantly enriched for follicle development and oocyte meiosis-associated biological processes, including the cell cycle and DNA repair (Fig. 4F and Table S3). Overall, these results suggest that METTL3, an m^6^A methyltransferase, can maintain oocyte development by stabilizing functional maternal mRNAs in the GV stage.

### IGF2BPs regulate the maternal mRNA abundance during oogenesis

IGF2 mRNA-binding proteins 1, 2, and 3 (IGF2BP1, IGF2BP2, and IGF2BP3, respectively) have been identified as a distinct family of m^6^A readers mediating mRNA stabilization [16]. Recently, IGF2BP2 and IGF2BP3 have been validated to maintain maternal mRNA abundance for early embryo development in mouse and zebrafish, respectively [36, 37]. As m^6^A-marked maternal mRNAs in GV stage oocytes are preferentially downregulated upon *Mettl3* depletion, we hypothesized that the change in maternal mRNA abundance in GV stage oocytes might be regulated by the IGF2BP family. Thus, we next investigated whether some of the m^6^A-marked maternal mRNAs could be directly targeted by the IGF2BP family. The expression of only *Igf2bp2* and *Igf2bp3* could be detected in GV stage oocytes (Fig. 5C), suggesting that *Igf2bp2* and *Igf2bp3* might be the main participants in the maintenance of maternal mRNA abundance in this stage. To explore the correlation between IGF2BP2/3 and m^6^A in GV stage oocytes, we downloaded public data on IGF2BP2/3 peaks [50] detected in human embryonic stem cells (hESCs) with eCLIP and converted them to mm10 genomic coordinates using liftOver. The distances between the IGF2BP2/3 and m^6^A peaks were calculated using BEDTools. The results showed that the distance between peaks was significantly closer than the shuffled background, especially for IGF2BP3 (Fig. 5D), suggesting that the region with m^6^A modification could be accessible to IGF2BP2 and even more easily accessible to IGF2BP3. Furthermore, we identified 617 (56.2%) downregulated m^6^A-modified maternal mRNAs targeted by IGF2BP2 or IGF2BP3 (Fig. 5E and Table S4). Among them were a group of transcripts that are responsible for oocyte meiosis and DNA repair (Fig. 5F). Collectively, these data indicate that IGF2BP2 and especially IGF2BP3, as m^6^A readers, might participate in stabilizing oogenesis-related maternal mRNAs involved in meiosis and DNA repair.

### METTL3 participates in oocyte maturation by regulating m^6^A modification of *Itsn2*

**Fig. 6:**
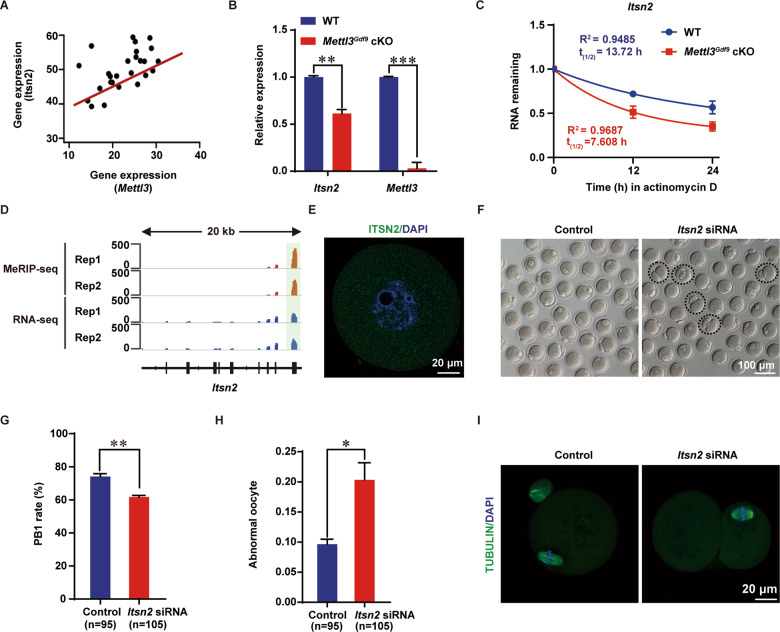
METTL3 regulates oocyte maturation by targeting m^6^A modification on *Itsn2*. **A** Scatter plot showing the Pearson correlation of RNA abundance between *Itsn2* and *Mettl3*. **B** qRT-PCR analysis of *Itsn2* and *Mettl3* mRNA levels in oocytes from 3-week-old WT and *Mettl3*^*Gdf9*^ cKO mice. The relative mRNA levels of *Itsn2* and *Mettl3* in WT oocytes were set to 1.0. **p < 0.01; ***p < 0.001 by two-tailed Student’s *t*-test. Data represent the mean ± SEM (*n* = 3). **C q**RT-PCR analysis of *Itsn2* mRNA stability in growing oocytes of WT and *Mettl3*^*Gdf9*^ cKO mice. Data were presented as means ± SEM, *n* = 3. **D** Integrated genomics viewer (IGV) plot showing the m^6^A peak across *Itsn2* mRNA transcript; the green box represents the m^6^A peak identified by MeRIP-seq. **E** Confocal immunofluorescence staining for ITSN2 (green) in oocytes. DAPI was used to stain nuclei (blue). Scale bar, 20 μm. **F** Images of control siRNA (Control) and *Itsn2* siRNA microinjected oocytes. Dashed circles indicate oocytes with big polar body or symmetrical division. Scale bar, 100 μm. **G**, **H** Rates of PB1 formation and abnormal oocytes after *Itsn2* siRNA and control siRNA (Control) microinjection. Control siRNA microinjected oocytes, *n* = 95; *Itsn2* siRNA microinjected oocytes, *n* = 105. *p < 0.05; **p < 0.01 by two-tailed Student’s *t*-test. Data represent the mean ± SEM (*n* = 3). **I** Confocal immunofluorescence staining with α-Tubulin antibody (green) and DAPI (blue) for *Itsn2* siRNA and control siRNA microinjected oocytes. Scale bar, 20 μm.

To elucidate the molecular mechanism of *Mettl3* in oocyte development, we compared the transcripts whose abundance positively correlated with *Mettl3* in the GV stage with those of 617 IGF2BP2/3 binding targets that contained m^6^A peaks and were downregulated upon *Mettl3* depletion. We found that five oocyte meiosis-associated transcripts (*Itsn2, Spire1, Myt1, Pias1*, and *Cdc42bpa*) and five DNA repair-associated transcripts (*Brca1, Ercc6l, Palb2, Brca2*, and *Mcm9*) were potentially regulated by METTL3 and IGF2BP2/3 via m^6^A modification (Fig. 5F and S2B). Among these potential targets, we found the homologs of *Itsn2*, S*pire1*, P*ias1*, E*rcc6l*, and B*rca2* are also targeted by IGF2BP3 using RIP-seq at the sphere stage of zebrafish [36] (Fig. S2C), indicating IGF2BP3 could regulate the fate of these maternal mRNAs at different developmental stages across species. As reported previously, the *Itsn2* gene encodes an adapter protein involved in microtubule formation and signal transduction and participates in meiosis during oocyte maturation [51]. Furtherly, we found that the abundance of *Itsn2* was significantly positively correlated with that of *Mettl3* at the GV stage (Fig. 6A). Next, qRT-PCR was performed, and the results validated the significant downregulation of *Itsn2* mRNA upon *Mettl3* deletion in the GV stage (Fig. 6B).

**Fig. 7:**
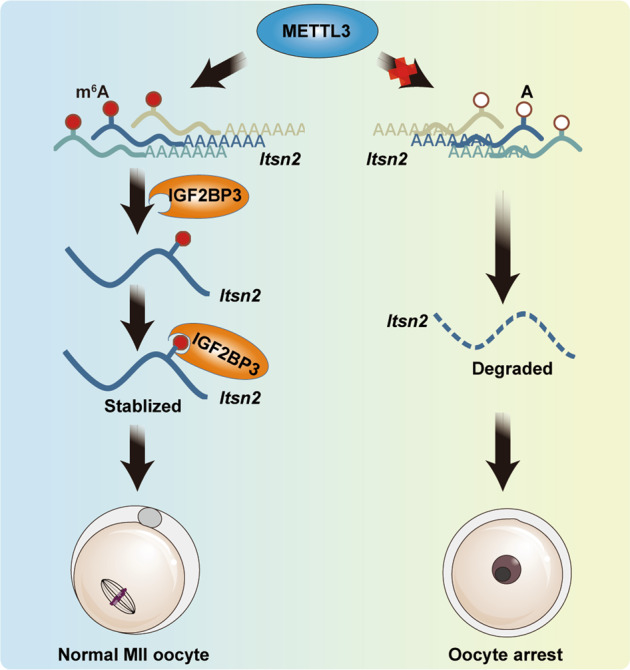
Schematic model for the regulatory landscape of the METTL3/IGF2BP3-m^6^A-*Itsn2* signaling axis in the promotion of oocyte development. In WT mouse oocytes, *Itsn2* mRNA with m^6^A could be recognized by IGF2BP3 to furtherly enhance its stability and promote oocyte meiotic maturation. However, in *Mettl3*^*Gdf9*^ cKO mouse oocytes, *Itsn2* mRNA without m^6^A could be degraded after losing IGF2BP3 protection, leading to oocyte meiotic maturation failure.

Next, we examined the stability of *Itsn2* mRNA and found a shortened mRNA half-life of *Itsn2* in *Mettl3*^*Gdf9*^ cKO growing oocytes compared with WT oocytes (Fig. 6C), indicating that m^6^A modification may promote *Itsn2* mRNA stability. In addition, our MeRIP-seq data and public IGF2BP3 RIP-seq data showed that there was only one METTL3-mediated m^6^A peak located in the CDS of the *Itsn2* transcript that might be recognized by IGF2BP3 (Figs. 6D, 5F, and S2C), indicating that IGF2BP3 is a potential cofactor for *Itsn2* abundance maintenance. Moreover, the immunofluorescence results showed that ITSN2 was expressed in the nucleus and cytoplasm of GV oocytes (Fig. 6E). Then, we screened three pairs of *Itsn2* candidate siRNAs for knockdown of *Itsn2* in oocytes, and we selected the most efficient siRNA, siRNA-1, for subsequent functional verification (Fig. S3A, B and Table S5). After injection of siRNA-1 for *Itsn2* knockdown, we found that the rate of polar body exclusion decreased significantly, and some abnormal oocytes showed a large polar body or symmetrical division (Fig. 6F–I). Collectively, the results illustrate that the m^6^A methyltransferase METTL3 participates in posttranscriptional regulation of *Itsn2* stability in oocytes, which is essential for oocyte maturation and meiotic divisions (Fig. 7).

## Discussion

METTL3, the core methyltransferase subunit, has been demonstrated to regulate postimplantation development [43], maintenance of the embryonic stem cell pluripotency network [52, 53], and spermatogenesis [38–40]. However, because its knockout in mice causes early embryonic lethality [43], the in vivo functions of *Mettl3* in female reproduction remain unknown. Our findings indicated that METTL3 was highly expressed during follicle growth and oocyte maturation (Fig. 1A, B). We then generated mice with oocyte-specific knockout of *Mettl3* at the primordial follicle stage to explore the potential role of *Mettl3* in the female germline. Interestingly, *Mettl3* knockout at the primordial follicle stage did not affect the survival of the primordial follicle until 6 weeks (Fig. 2A, B), the phenotype is more manifested in oocyte maturation at this stage (Fig. 3). Until 8 weeks, *Mettl3*^*Gdf9*^ cKO female mice displayed a severe defect in follicle development, which showed more primary follicles and fewer secondary follicles (Fig. 2A). In addition, it has been reported that METTL3 plays an important role in regulating unrepaired DSBs and genome instability by modulating DNA-RNA hybrid accumulation [54]. We found more DSBs are produced in the secondary follicles upon *Mettl3*^Gdf9^ cKO (Fig. 2C, D), indicating METTL3-deficient oocytes may also undergo apoptosis. Moreover, it has been further verified by terminal deoxynucleotidyl transferase dUTP nick end labeling (TUNEL) staining (Fig. 2E, F). Therefore, we speculate that might also be a potential mechanism to regulate oocyte DSBs and apoptosis in the *Mettl3*^*Gdf9*^ cKO mice. Thus, our study shows that METTL3 is not necessary for the transition of resting primordial follicles to activated growing follicles, but participates in follicle and oocyte development and plays an important role in female reproduction.

As reported previously, m^6^A regulatory enzymes can participate in the meiotic maturation of oocytes by affecting associated RNA metabolic processes. For example, the writer protein KIAA1429 and the reader protein YTHDC1 have been reported to regulate oocyte meiotic maturation by altering RNA alternative splicing [34, 35], and the reader protein YTHDF2 influences oocyte maturation by regulating maternal mRNA degradation [29, 55]. However, the fates of only certain maternal mRNAs can be influenced by these proteins, and the proportion of maternal mRNAs with m^6^A modification in oocytes is still unclear. Although *Mettl3* was knockout from the primordial follicle stage, the phenotype began to appear at later follicle developmental stages (Fig. 3). So in this work, we performed, for the first time, MeRIP-seq using low-input materials from GV oocytes based on a previously described protocol with some modifications [56]. According to the sequencing data, we identified 3256 (45.8%) maternal RNAs expressed in GV oocytes that contained m^6^A modifications, suggesting that the function of m^6^A in oocytes has been underestimated. Through a combination of RNA-seq and MeRIP-seq data, we found that m^6^A-modified transcripts showed significantly lower expression upon *Mettl3* deletion than transcripts without m^6^A (Fig. 4E). We identified 1098 (53.5%) downregulated transcripts with m^6^A modification that were enriched for meiosis and DNA repair (Figs. 5B, 4F). Consistently, our results showed that the process of oocyte meiotic maturation was defective upon *Mettl3* deletion, suggesting that meiosis-associated maternal transcripts with m^6^A modification are preferentially stabilized in the GV stage (Fig. 4E, F). As reported previously, IGF2BPs have been validated to bind to the maternal mRNA and promote its stability during early embryo development in mouse and zebrafish, respectively [36, 37]. Thus, we hypothesized that IGF2BPs might be the main cofactor to regulate the fate of maternal mRNAs with m^6^A in follicle and oocyte development. Furtherly, we found 617 (56.2%) downregulated transcripts with m^6^A modification that were targeted by IGF2BP2 or IGF2BP3 (Fig. 5E), including meiosis-associated genes, such as *Itsn2*, *Cdc42bpa*, *Spire1*, Myt1, and *Pias1* (Fig. 5F). *Itsn2* has been reported to be an adapter protein that regulates oocyte meiotic resumption through the Cdc42 pathway [51]. *Cdc42bpa* is a Cdc42 downstream effector that also regulates meiotic oocytes through the Cdc42 signaling pathway [57]. *Spire1* has been reported to drive asymmetric oocyte division by cooperating with Formin-2 [58]. *Myt1* is a transcription factor that has been identified as a trigger during oocyte meiosis [59]. *Pias1* is a member of the SUMO pathway, which is important for the maintenance of centromeric cohesion [60]. In support of these previous findings and based on its significant positive correlation with *Mettl3* in the GV stage (Fig. 6A) and that it could be targeted by IGF2BP3 at early embryo development (Fig. S2C), we focused on *Itsn2*. Specifically, we knocked down *Itsn2* by microinjecting siRNA into GV oocytes. Taken together, our results showed that in WT mouse oocytes, m^6^A modifications on *Itsn2* mRNA might be recognized by IGF2BP3, which further enhanced the stability of the mRNA, consequently promoting oocyte meiotic maturation. However, in *Mettl3*^*Gdf9*^ cKO mouse oocytes, *Itsn2* mRNA could not be recognized by IGF2BP3; consequently, degradation of the mRNA was promoted, leading to oocyte meiotic maturation failure (Fig. 7).

In conclusion, our results show that m^6^A serves as a critical regulator to control the stability of oocyte meiotic maturation-related transcripts and that the precise effect of m^6^A on transcript stability might depend on different reader proteins. In addition to playing a regulatory role in mRNA stability, m^6^A may also participate in oocyte maturation by affecting other RNA metabolic processes. Exploration of the detailed mechanisms of this possible role will be of great interest in the future.

## Materials And methods

### Mice

The conditional mutant alleles for *Mettl3* (hereafter referred to as *Mettl3*^*flox/flox*^) were kindly provided by Dr. Ming-Han Tong at Shanghai Institute of Biochemistry and Cell Biology [40]. *Gdf9*-Cre transgenic mice were a gift from Dr. Qing-Yuan Sun at Guangdong Second Provincial General Hospital [61]. All mice described above were maintained on the C57BL/6 J background. Mice lacking *Mettl3* in oocytes (referred to as *Mettl3*^*Gdf9*^ cKO) were generated by crossing *Mettl3*^*flox/flox*^ mice with *Gdf9*-Cre mice. The *Mettl3*^*flox/flox*^ female mice were used as the control group (referred to as WT). For the fertility test, six pairs of 6 weeks *Mettl3*^*flox/flox*^ and *Mettl3*^*Gdf9*^ cKO female mice were randomly selected and continually mated to *Mettl3*^*flox/flox*^ male mice which have been confirmed fertility for 5 months. The number of pups and litter size from each female was recorded. Primers for PCR genotyping were listed in Table S6. All animal experiments were done in accordance with the guidelines from the Animal Care and Use Committee of China Agricultural University.

### Oocyte and zygote collection

To obtain fully-grown GV oocytes, 6–8 weeks of females were injected with 5 IU PMSG (Ningbo No.2 hormone factory, Zhejiang, China). After 44–48 h, GV oocytes were collected by puncturing the ovarian follicle and released with microcapillary pipettes in M2 media (M-7197, Sigma-Aldrich, Germany). For MII oocyte collection, mice were injected with PMSG as above and after 46–48 h with 5 IU of human chorionic gonadotropin (hCG) (Ningbo No.2 hormone factory, Zhejiang, China). MII oocytes were collected from the oviducts 14–16 h post hCG injection by digestion in M2 medium with hyaluronidase (MR-051-F, Millipore, USA). The detailed procedure to get meiotically incompetent PD5 and PD12 oocytes was described previously [25, 62].

To obtain zygotes, female mice were mated with male mice with known fertility. Successful mating was confirmed by the presence of vaginal plugs. Zygotes were isolated from the oviduct of plugged females. The zygotes were cultured in KSOM medium (MR-107-D, Millipore, USA) and cultured in a 5% CO_2_ incubator.

### Oocyte microinjection

GV oocytes were collected from 6–8 weeks CD-1 mice (Beijing Vital River Laboratory Animal Technology Co., Ltd) follow the same procedure above. For the siRNAs injection experiment, *Itsn2* siRNAs were designed and synthesized by GenePharma (Shanghai, China) and were dissolved in RNase-free water to a final concentration of 20 mM. Approximately 5 pl of the siRNAs were microinjected into the cytoplasm of fully-grown GV oocytes in M2 medium serum. After injection, oocytes were cultured for 20–24 h in 200 μM IBMX (I5879, Sigma-Aldrich, Germany) containing medium to maintain GV arrest, and then the oocytes were either collected for assessing the knockdown efficiency by qRT-PCR and western blot analysis or alternatively washed from the IBMX and cultured in maturation medium for 14 h for scoring the progress of meiosis. Sequences of siRNAs were listed in Table S5.

### Quantification of ovarian follicles and histological analysis

The ovaries were fixed in 4% paraformaldehyde overnight at 4 °C, dehydrated ethanol series, and embedded in paraffin. To count the numbers of follicles, paraffin-embedded ovaries were serially sectioned at the 5-μm thickness and stained with hematoxylin for histological analyses. Primordial, primary, secondary, and antral follicles were counted in every fifth section of an ovary. In each section, follicles that contained oocytes with clearly visible nuclei were scored and the cumulative number of follicles was multiplied by a correction factor of 5 to represent the estimated number of total follicles in an ovary. A double-blind experiment was performed for the quantification of ovarian follicles.

### Immunohistochemistry and immunofluorescence

For immunohistochemistry (IHC) and immunofluorescence on ovary sections, ovaries were fixed, embedded, and sectioned follow the same procedure above. Sections then were boiled in 10 mM sodium citrate buffer (pH 6.0) for 18 min for antigen retrieval, cooled down in ice for 20 min, and washed in PBS with 0.1% Triton X-100. The following steps for IHC were under the instruction from the SP Rabbit or Mouse HRP Kit (ZSGB-BIO, China). The primary antibodies for IHC include Anti-METTL3 antibody (ab195352, 1:500, Abcam, UK), Anti-DDX4/MVH antibody (Ab27591, 1:500, Abcam, UK). The following steps for immunofluorescence on ovary sections was performed as previously described [40]. The primary antibodies for immunofluorescence include the Anti-γH2AX antibody (ab11174, 1:500, Abcam, UK). An apoptotic signal was detected using DeadEnd™ Fluorometric TUNEL System (G3250, Promega, USA) according to the manufacturer’s instructions.

For immunofluorescence on oocytes, oocytes were fixed in 4% paraformaldehyde (PFA) for 30 min at room temperature, and then treated with 0.25% Triton X-100 in PBS for 15 min. After blocking with 1% BSA in PBS for 60 min, oocytes were incubated with primary antibodies: FITC-α-Tubulin antibody (F2168, 1:100, Sigma-Aldrich, USA), anti-intersectin2 antibody (NBP1-71833, 1:1000, NOVUS, USA), anti-METTL3 antibody (ab195352, 1:500, Abcam, UK) at 4 °C overnight, followed by incubation with secondary antibodies for 1 h at room temperature. After washing three times with 0.1% BSA in PBS, oocytes were counterstained with Mounting Medium with DAPI (ab104139, Abcam, UK), and then analyzed by confocal microscopy A1 (Nikon, Japan).

### RNA isolation and qRT-PCR

Total RNA was extracted from oocytes samples using Trizol reagent (15596018, Invitrogen, USA), and cDNA was generated using the 5X All-In-One RT Master Mix (G490, Abm, USA). Quantitative real-time PCR using 2× RealStar Green Power Mixture (A311, GenStar, China) was performed using a real-time PCR system (Roche LightCycler 96^®^, Germany). Primer sequences are listed in Table S7.

### RNA stability analysis

Primary (25–35 μm) and secondary (35–65 μm) oocytes from 3-week-old WT and *Mettl3*^*Gdf9*^ cKO ovaries by enzymatic digestion follow the same procedure above [25, 62]. RNA stability analysis was performed as previously described with some modifications [63, 64]. The oocytes were collected at 0, 12, 24 h after in vitro culture with Actinomycin D (10 μg/ml, SBR00013, Sigma, Germany) treatment. Total RNA isolation and qRT-PCR were performed as above and β-actin was used as a loading control for normalization. Plot the relative expression of RNA at each time point relative to *t* = 0 and then calculate the mRNA decay rate by nonlinear regression curve fitting (one phase decay) using GraphPad Prism (version 8). Primer sequences are listed in Table S7.

### Hormone assays

Twelve to 20 weeks old *Mettl3*^*Gdf9*^ cKO and WT female mice were sacrificed in order to measure FSH and LH levels. Serum samples were collected as described previously [48, 61] and stored at −80 °C until measurement. The assays were using ELISA Kit for Follicle-Stimulating Hormone (CEA830Mu, CLOUD-CLONE, China) and ELISA Kit for Luteinizing Hormone (CEA441Mu, CLOUD-CLONE, China) according to the manufacturer’s instructions.

### RNA-seq

Ten mouse GV oocytes were collected in lysis buffer and subjected to first-strand cDNA synthesis using SMARTer^®^ PCR Synthesis Kit (634925, Clontech, Japan). The cDNA products were analyzed by Agilent 2100 bioanalyzer and were fragmented with sonication and subjected to library construction using TruSeq^®^ Nano DNA Library Prep kit (FC-121–403, Illumina, USA) according to the manufacturer’s instructions. Sequencing was performed on an Illumina HiSeq X-ten sequencing system.

### Western blot

About 100 fully-grown GV oocytes were lysed in 2× Laemmli Sample Buffer (1610737, Bio-Rad, USA) with protease inhibitor. Oocyte lysates were heated at 99.9 °C for 5 min and the denatured protein samples were separated by SDS-PAGE gel and transferred to PVDF membranes (IPVH00010, Millipore, Germany). Membranes were blocked with 5% nonfat milk prepared in Tris-buffer saline-plus 0.1% Tween-20 (TBST) at room temperature for 1 h and then incubated with primary antibodies overnight at 4 °C, followed by the treatment with Goat anti-Rabbit-HRP (BE0106, 1:5000, Easybio, China) for 1 h on the next day. After washing with TBST three times, the blotted membranes were exposed with SuperSignal West Dura Extended Duration Substrate (34075, Thermo, USA). The following primary antibodies were used for western blot: anti-METTL3 antibody (ab195352, 1:500, Abcam, UK), ITSN2 antibody (NBP1-71833, 1:1000, NOVUS, USA), and GAPDH antibody (2118 L, 1:5000, CST, USA).

### m^6^A-MeRIP-seq

m^6^A-MeRIP using low-input materials was performed based on a previously described protocol [55] with some modifications. Briefly, total RNA from about 2500 mouse GV oocytes from 5 weeks C57BL/6 J mice (Beijing Vital River Laboratory Animal Technology Co., Ltd) was first randomly fragmented to ~200 nt with RNA fragmentation reagents (AM8740, Thermo, USA), and then incubated with the protein A beads (10001D, Thermo, USA) coupled with anti-m^6^A polyclonal antibody (ABE572, Millipore, USA) in IPP buffer (150 mM NaCl, 10 mM Tris-HCl, pH 7.4, 0.1% NP-40, 0.4 U/μl RNasin). After immunoprecipitation, the RNA reaction mixture was washed twice in 1 ml of IP buffer, twice in 1 ml of low-salt IP buffer (50 mM NaCl, 10 mM Tris-HCl, pH 7.4, 0.1% IGEPAL^®^ CA-630 (I8896, Sigma-Aldrich, Germany) in nuclease-free H_2_O), and twice in 1 ml of high-salt IP buffer (500 mM NaCl, 10 mM Tris-HCl, pH 7.4, 0.1% IGEPAL CA-630 in nuclease-free H_2_O) for 5 min each at 4 °C. After extensive washing, the m^6^A-enriched RNA fragments were eluted from the beads by proteinase K digestion followed by phenol-chloroform extraction and ethanol precipitation. The purified RNA was subjected to library construction using the SMARTer Stranded Total RNA-Seq Kit v2 (634413, Clontech, Japan) according to the manufacturer’s instructions. Sequencing was performed on an Illumina HiSeq X-ten sequencing system.

### Sequencing data analysis

General preprocessing of reads: the MeRIP-seq of control, the RNA-seq of WT, and *Mettl3*^*Gdf9*^ cKO treatment for GV oocyte were performed using Illumina HiSeq platform with paired-end 150 bp read length. Adapter sequences were trimmed off for all raw reads using the Cutadapt (version 1.18). Especially, for MeRIP-seq that generated using SMARTer Stranded Total RNA-Seq Kit version 2, the first three nucleotides of the second sequencing read which derived from the SMART adapter was trimmed using Cutadapt with parameter “-U 3”. Reads with length less than 35 nt or contained an ambiguous nucleotide were discarded by Trimmomatic (version 0.36). The remaining reads were aligned to the mm10 by HISAT2 (version 2.0.5). To minimize the rate of false positives, only uniquely mapped reads with −q ≥ 20 were kept for the subsequent analysis for each sample.

For MeRIP-seq, the whole-genome m^6^A-enriched peaks were identified using MACS (version 2.1.4) with the corresponding input sample as a control. MACS was used with parameters “--nomodel, --keep-dup all and -g mm”. Peaks with FDR value <0.05 was annotated based on Ensembl (release 68) gene annotation information by applying BEDTools’ intersectBed (version 2.16.2). Only peaks located in the GV oocyte-expressed gene and annotated as TSS (transcription start sequence), 5′UTR, CDS, stop codon, and 3′UTR were used for the subsequent analysis.

For RNA-seq, the number of reads mapped to each gene was counted using the HTSeq (version 0.6.0), with parameter ‘--mode = union and --stranded = no’. RPKM (reads per kilobase of exon model per million mapped reads) was calculated for each gene using an in-house Rscript. Only transcripts with the average RPKM >3 for WT samples were regarded as expressing in GV oocyte. The R package DESeq2 was used for differential expression analysis (fold change cutoff = 1.2, *p* value cutoff = 0.05, and the average RPKM >3 for WT). Cumulative distribution analysis of RNA-seq log_2_ fold changes for expressed transcripts between control and *Mettl3*^*Gdf9*^ cKO treatment was performed in R using the ecdf function. Groups were defined as m^6^A (MeRIP-seq FDR <0.05) or non-m^6^A (the remainder of the transcripts). The significance of the difference between the cumulative distribution curves was calculated by the Kolmogorov–Smirnov test.

### Motif statistics analysis within m^6^A peaks

Motif analysis for m^6^A peaks were identified by HOMER (version 4.7). The sequence of peaks located in TSS, 5′UTR, CDS, stop codon, and 3′UTR for mRNA were extracted as the target sequences and background sequences were obtained by randomly shuffling peaks upon total mRNAs on the genome using BEDTools’ shuffleBed.

### m^6^A reader analysis

IGF2BP2/3 binding peaks identified by eCLIP for hESC were downloaded from GSE78509. Human coordinates were converted to mm10 using UCSC tools’ liftOver. Distance between IGF2BP2/3 peaks by eCLIP and m^6^A peaks identified in this study was calculated using BEDTools. Background were obtained by randomly shuffling IGF2BP2/3 peaks using BEDTools’ shuffleBed. Expressed transcripts with m^6^A peaks, targeted by IGF2BP2/3, were obtained using the union genes identified by eCLIP (GSE78509) [50] and RIP-seq (GSE90639) [16].

### Gene set enrichment analysis

Functional enrichment analysis of KEGG pathways documented in Gene Ontology were performed using DAVID (https://david.ncifcrf.gov). KEGG terms with *p* value <0.05 were determined to be statistically significant.

### Quantification and statistical analysis

All the experimental data was replicated at least in two independent experiments. Data in figures were expressed as mean ± SEM unless otherwise stated. Replicates in different experiments were stated in corresponding Figure legends. Statistically significant differences between different groups were evaluated by Student’s *t*-test, two-sided Kolmogorov–Smirnov test, Chisq-test. *p* < 0.05 were considered as statistically significant. The significance of Pearson correlation (Figs. 3F and 4A) was evaluated by the R program. Statistical significance in other experiments was performed by Graphpad Prism (version 8). For microscope images, n generally refers to the total number of oocytes and embryos.

## Supplementary information

Figure S1

Figure S2

Figure S3

Supplementary figure and table legends

Table-S1

Table-S2

Table-S3

Table-S4

Table-S5

Table-S6

Table-S7

## Data Availability

The RNA-seq and MeRIP-seq data have been deposited into National Genomics Data Center (https://bigd.big.ac.cn) with accession number: PRJCA003168.

## Code availability

The computer code used during the current study is available from the corresponding authors on reasonable request.
