# Supplementary figures and images for: METTL3-mediated mRNA N6-methyladenosine is required for oocyte and follicle development in mice
Source: Cell Death Dis. 2021 Oct 23;12(11):989. doi: 10.1038/s41419-021-04272-9 (PMC8542036; doi:10.1038/s41419-021-04272-9)

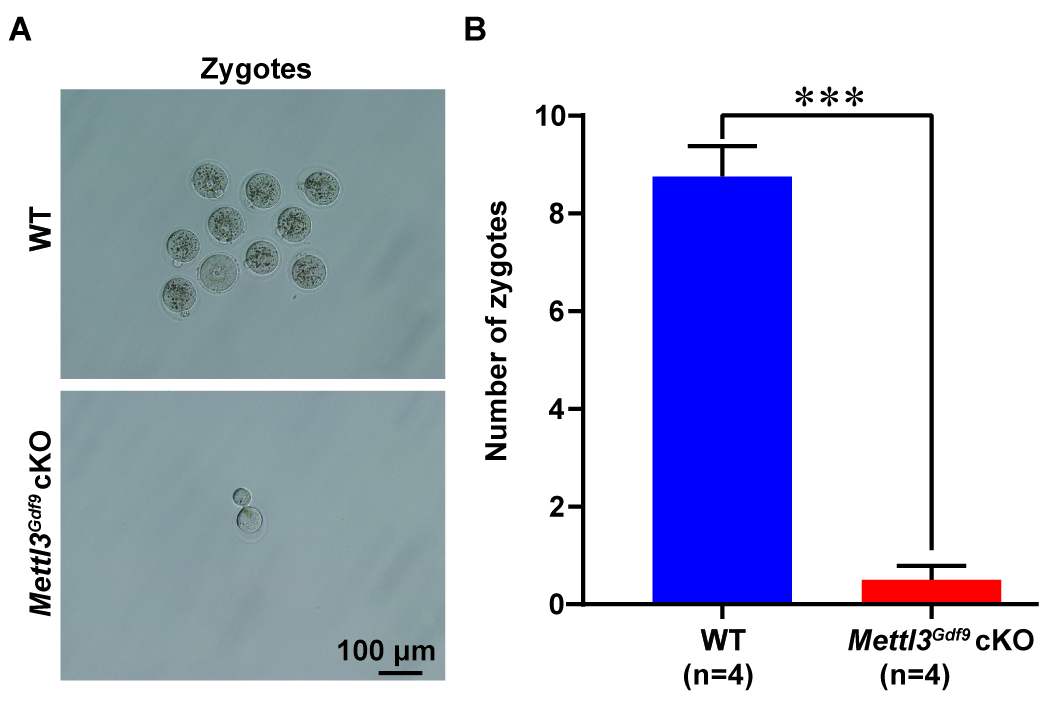

Supplement: Supplementary file 1 — Figure S1 [file 41419_2021_4272_MOESM1_ESM.tif]

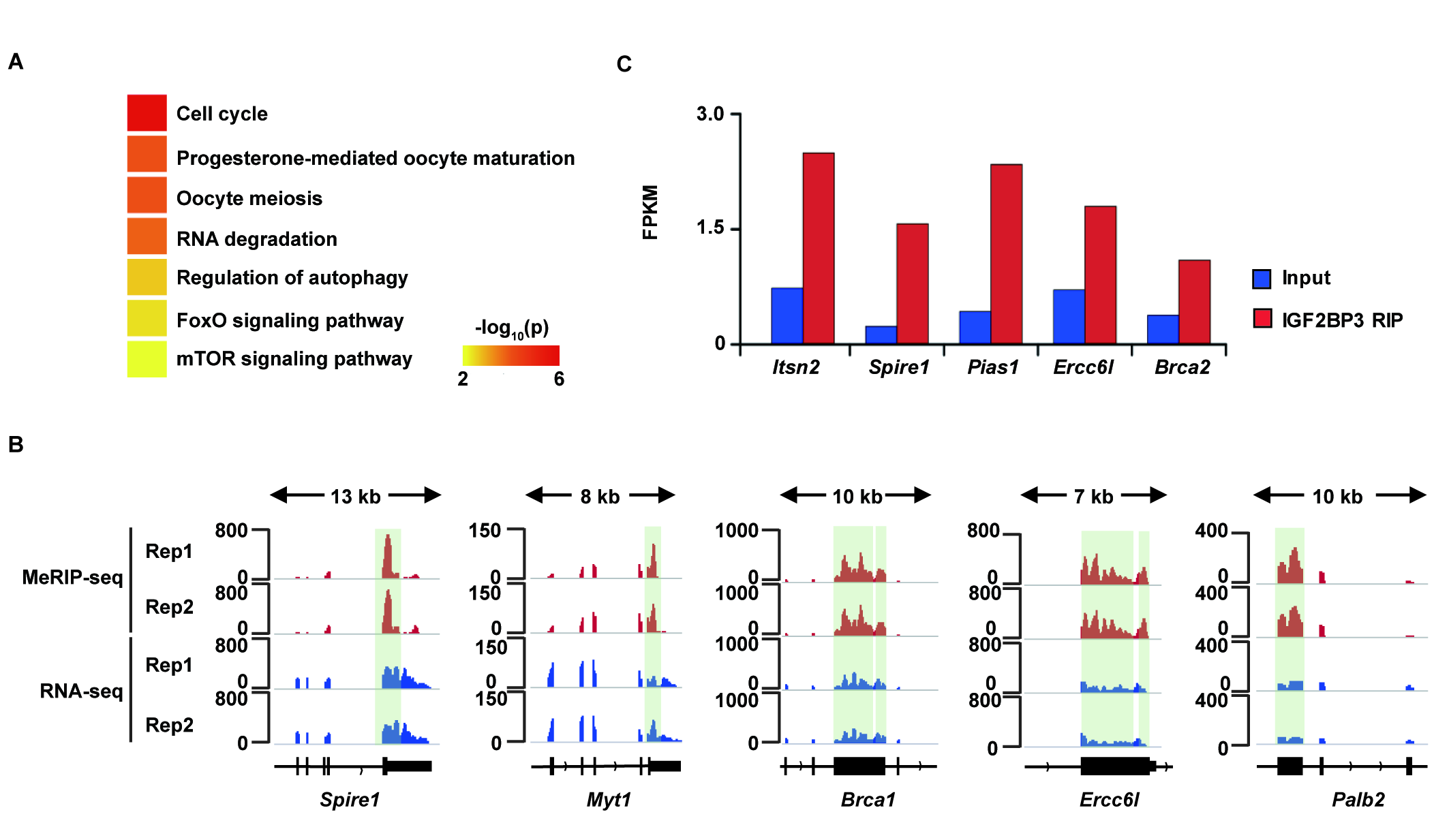

Supplement: Supplementary file 2 — Figure S2 [file 41419_2021_4272_MOESM2_ESM.tif]

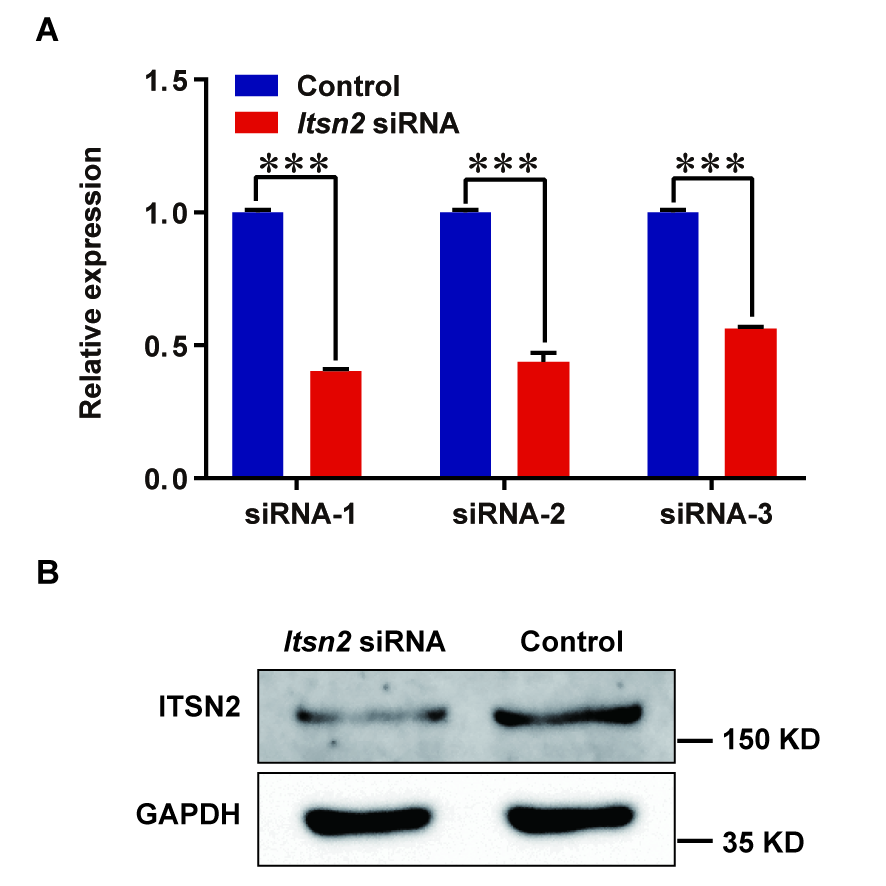

Supplement: Supplementary file 3 — Figure S3 [file 41419_2021_4272_MOESM3_ESM.tif]
