# Supplementary material for: METTL3-mediated mRNA N6-methyladenosine is required for oocyte and follicle development in mice
Source: Cell Death Dis. 2021 Oct 23;12(11):989. doi: 10.1038/s41419-021-04272-9 (PMC8542036; doi:10.1038/s41419-021-04272-9)
Supplement: Supplementary file 4 — Supplementary figure and table legends [file 41419_2021_4272_MOESM4_ESM.docx]

**SUPPLEMENTARY FIGURE AND TABLE LEGENDS**

**Fig. S1**

**A-B** Representative images and the number of zygotes collected from 8-week-old WT and *Mettl3^Gdf9^* cKO mice after natural ovulation. Scale bar, 100 μm. ***, p < 0.001 by two-tailed Student’s t-test. Data represent the mean ± SEM (n=4).

**Fig. S2**

**A** Heatmap showing significantly enriched KEGG pathways regulated by downregulated transcripts upon *Mettl3^Gdf9^* cKO.

**B** Integrated Genomics Viewer (IGV) plot showing the m^6^A peaks across *Spire*, *Myt1*, *Brca1*, *Ercc6* and *Palb2* transcripts whose abundance levels are positively correlated with that of *Mettl3* (R > 0.4). The green box represents the m^6^A peak identified by MeRIP-seq.

**C** Bar plot showing the normalized read count (FPKM) of *Itsn2*, *Spire1*, *Pias1*, *Ercc6l* and *Brca2* in IgG-IP (blue) and IGF2BP3-HA-IP (red) samples, which determined by RIP-seq documented in NCBI BioProject under accession number PRJNA565584. Transcripts are defined as IGF2BP3 targets with FPKM > 1 in all IP samples and were upregulated >2-fold in HA-IP sample compared with IgG-IP sample.

**Fig. S3**

**A** Knockdown efficiency of 3 pairs of *Itsn2* siRNAs was confirmed by qRT-PCR. The relative mRNA level of *Itsn2* in WT oocytes was set to 1.0. ***, p < 0.001 by two-tailed Student’s *t*-test. Data represent the mean ± SEM (n = 3).

**B** Immunoblotting analysis of ITSN2 protein level in *Itsn2* siRNA and control siRNA microinjection oocytes. GAPDH was used as an internal control. One hundered germinal vesicle oocytes were used for each lane of the blots.

**Table S1.** Information of m^6^A peaks identified using MeRIP-seq in GV stage

**Table S2.** Information of differential expressed transcripts upon *Mettl3^Gdf9^* cKO

**Table S3.** KEGG pathways enriched by down-regulated genes upon *Mettl3* depletion or genes with m^6^A modification

**Table S4.** Information of IGF2BPs targets which have m^6^A modifications and are down-regulated upon *Mettl3^Gdf9^* cKO

**Table S5.** siRNA squences

**Table S6.** Primers for genotyping

**Table S7.** Primers for qRT-PCR
